# A75 COLONOSCOPY COLORECTAL CANCER DETECTION - HOW DOES IT VARY BY INDICATION, SETTING AND PATIENT DEMOGRAPHIC?

**DOI:** 10.1093/jcag/gwac036.075

**Published:** 2023-03-07

**Authors:** C Dube, B R McCurdy, J Sacco, J Gao, K Silveira, T Karapetyan, Y Niu, J Tinmouth

**Affiliations:** 1 Medicine, The Ottawa Hospital, Ottawa; 2 Ontario Health - Cancer care Ontario; 3 Medicine, Sunnybrook Hospital, Toronto, Canada

## Abstract

**Background:**

Because of limited access to gastrointestinal (GI) endoscopy during the COVID-19 pandemic, there is a need to prioritize procedures to avoid negative health impacts from delays. Ontario Health (OH) has provided guidance to facilities to prioritize colonoscopies in people with an abnormal fecal immunochemical test (FIT) result, based on the high likelihood to detect invasive colorectal cancer (CRC) and recommends FIT for screening people at average risk of CRC and those with prior low-risk adenoma.

**Purpose:**

To measure the invasive CRC detection rate (CDR) of colonoscopies performed in Ontario by indication, setting, age, and sex over a 31-month period before and during the COVID-19 pandemic.

**Method:**

We calculated the CDRs among outpatients ages 18 and over who had colonoscopies performed in a hospital or private clinic setting from June 2019 to December 2021. We identified hospital colonoscopies from OH’s GI Endoscopy Data Submission Portal and clinic colonoscopies from Ontario Health Insurance Plan (OHIP) data (OHIP code E749A). Invasive CRC was identified from the Ontario Cancer Registry (OCR) as: ICD-O-3 codes C18.0, C18.2-C18.9, C19.9, C20.9, a morphology indicative of CRC, microscopically confirmed and with a pathology report. CRCs were included if diagnosed 7 days before and up to 183 days after colonoscopy. Colonoscopy volumes and CDRs were stratified by age and sex (all volumes), and, for hospital colonoscopies, by indication.

**Result(s):**

During the study period, 984,109 colonoscopies were performed (638,900 in hospitals; 345,209 in clinics). Patients who had their colonoscopies in clinics were younger than those who had them in hospitals (Table 1). In both settings, colonoscopies were evenly distributed by sex. Overall, 12,021 CRCs were detected (CDR: 1.22%); 9,451 CRCs in hospitals (CDR: 1.48%), and 2,570 CRCs in clinics (CDR: 0.74%). CDRs at any age were lower in clinics as compared to hospitals. In hospitals, CDRs by colonoscopy indication were: 5.16% for FIT+, 1.93% in symptomatic patients, 0.52% in surveillance, 0.70% in average-risk screening, and 0.35% in screening due to family history. FIT+ colonoscopies accounted for the smallest proportion of colonoscopies (6.2%) but the 2^nd^ largest proportion of CRCs detected (Figure 1). Hospital-based CDR increased during the period of observation from 1.23% pre-pandemic (June-December 2019) to 1.55% during the pandemic (January-December 2021). Clinic CDR was 0.71% pre-pandemic and 0.75% during the pandemic.

**Image:**

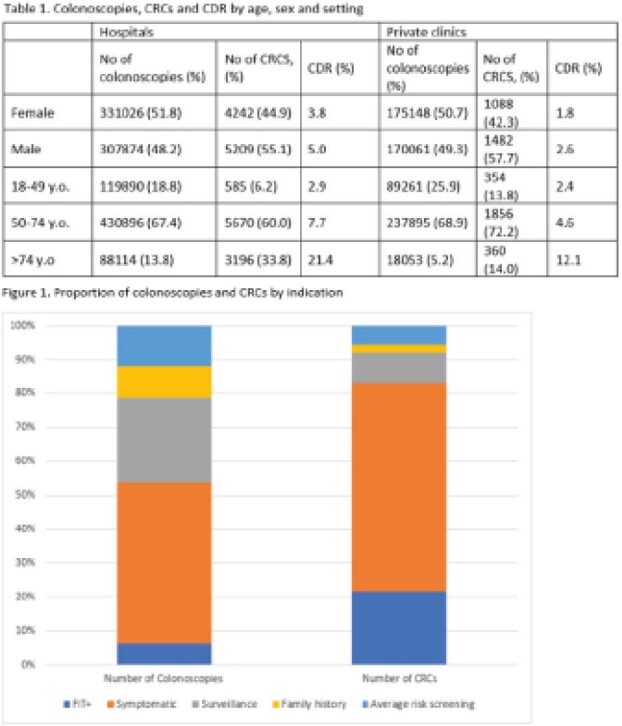

**Conclusion(s):**

In Ontario, colonoscopy yield (CDR) is highest in FIT+ patients; just over one in 20 colonoscopies will yield a diagnosis of CRC. In contrast, primary screening and surveillance indications have very low CRC yields. The overall yield of colonoscopies in clinics, for all age groups, is lower than in hospital setting. There was a slight increase in CDR during the pandemic compared to before the pandemic, in both settings.

**Please acknowledge all funding agencies by checking the applicable boxes below:**

Other

**Please indicate your source of funding;:**

Ontario Health - Cancer Care Ontario

**Disclosure of Interest:**

None Declared

